# Catalytic His-loop flexibility drives high activity in hyperthermophilic esterase EstE1 while preserving structural stability

**DOI:** 10.1128/spectrum.01003-25

**Published:** 2025-08-14

**Authors:** Khang Nguyen, ChangWoo Lee

**Affiliations:** 1 Department of Biomedical Science, Daegu University 37975, Gyeongsan, South Korea; 2 Center for Bio-Nanomaterials, Daegu University 37975, Gyeongsan, South Korea; Gujarat Biotechnology University, Gandhinagar, Gujarat, India

**Keywords:** catalytic His loop, conformational flexibility, active-site dynamics, esterase, thermal stability, temperature adaptation

## Abstract

EstE1, a hyperthermophilic esterase, exhibits high catalytic activity despite its rigid structure. This study investigates how the catalytic His loop modulates enzyme activity in EstE1 and its mesophilic counterpart, rPPE. Mutations were introduced at Gly282 in EstE1 (G282N and G282Q) to promote hydrogen bond formation and at Asp287 in rPPE (D287G and D287E) to disrupt or strengthen loop-stabilizing interactions. In EstE1, both G282N and G282Q reduced activity. Wild-type (WT) EstE1 displayed a gradual, temperature-dependent fluorescence decrease from 4°C to 90°C, partly attributed to Tyr182 located in the active-site wall. The G282N mutant exhibited similar fluorescence between 4°C and 25°C and increased rigidity in acrylamide quenching, consistent with hydrogen bond formation, but demonstrated a time-dependent loss in residual activity. In contrast, G282Q failed to form the hydrogen bond and exhibited greater flexibility than WT. In rPPE, increased loop flexibility in D287G enhanced activity without compromising active-site stability. The D287E mutant, which maintained activity similar to WT, formed a stronger hydrogen bond and improved both stability and substrate affinity. These findings suggest that Gly282 promotes His-loop flexibility, which is essential for EstE1’s high activity at elevated temperatures, whereas rPPE relies on hydrogen bonding to stabilize the His loop, limiting activity but preserving active-site stability. Overall, this study provides insights into engineering thermophilic enzymes with improved catalytic performance while maintaining structural stability.

**IMPORTANCE:**

Esterases, which commonly share the catalytic triad Ser-His-Asp, play essential roles in diverse biological processes by catalyzing ester bond formation and cleavage. EstE1 features a flexible catalytic His loop despite its overall rigid structure, whereas its mesophilic counterpart, rPPE, has a more flexible global structure but a rigid His loop stabilized by hydrogen bonding. Introducing a hydrogen bond into the His loop of EstE1 reduces activity and active-site stability, while disrupting this interaction in rPPE enhances activity without compromising stability. Fluorescence and quenching analyses further highlight distinct loop conformations and flexibility profiles among the different mutants. These findings illustrate how EstE1 achieves high activity at elevated temperatures by incorporating catalytic His-loop flexibility into its rigid scaffold. This mechanistic insight provides a valuable framework for engineering thermophilic enzymes with enhanced catalytic performance while preserving structural stability.

## INTRODUCTION

Carboxylesterases (EC 3.1.1.1) are widespread enzymes that hydrolyze a variety of substrates, including esters, amides, thioesters, and carbamates (1–4). Most belong to the α/β-hydrolase family, characterized by a central parallel β-sheet surrounded by α-helices, forming a distinct structural motif (3, 5). Their active site contains a substrate-binding pocket and a catalytic triad composed of Ser, His, and Asp/Glu (3, 5–8). The nucleophilic Ser, located at the base of this pocket, resides within a conserved GXSXG pentapeptide motif, and an oxyanion hole stabilizes the reaction intermediate during catalysis (6, 8–11). Hyperthermophilic esterases typically possess rigid structures, stabilized by strong intramolecular interactions and reduced cavity formation, enabling them to maintain structural integrity at high temperatures (8, 12–15). In contrast, enzymes adapted to lower temperatures generally exhibit greater flexibility, with reduced intramolecular interactions, extended loops, and more accessible active sites that facilitate efficient catalysis at lower temperatures by lowering the Gibbs free energy of activation (ΔG^‡^) (16, 17).

Notably, some hyperthermophilic esterases, such as EstE1 (from a thermal environmental sample) and AFEST (*Archaeoglobus fulgidus*), defy the conventional activity-stability trade-off. Despite their structural rigidity, they exhibit higher catalytic rates than mesophilic counterparts (15, 18–22). For example, EstE1, which hydrolyzes p-nitrophenyl (pNP) hexanoate with an optimal temperature of 70°C, shows a 212-fold higher catalytic rate than the mesophilic esterase rPPE (*Pseudomonas putida*), which hydrolyzes pNP acetate at its optimal temperature of 45°C (19). Similarly, AFEST exhibits a catalytic rate of 1,014 s^−1^ at 70°C with pNP hexanoate (22). These observations raise a fundamental question: how do hyperthermophilic esterases achieve such high catalytic activity while retaining structural stability?

**Fig 1:**
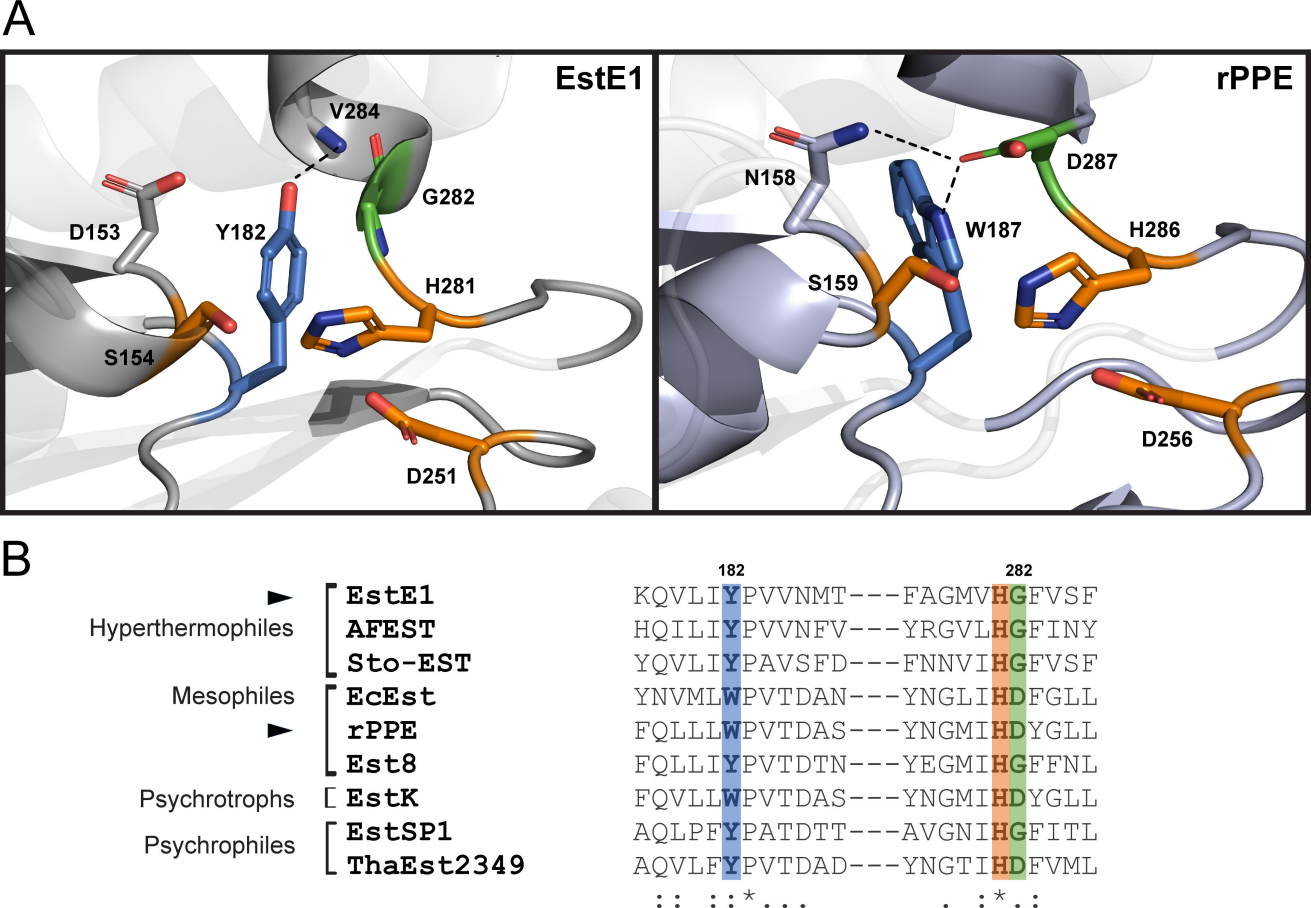
Crystal structures of EstE1 and rPPE and multiple sequence alignment of esterases from different thermal environments. (**A**) Active-site structures of EstE1 and rPPE. Catalytic triad residues are shown in orange, Gly282 (EstE1) and Asp287 (rPPE) in green, and aromatic residues in blue. (**B**) Multiple sequence alignment of esterases adapted to different habitat temperatures. **Hyperthermophilic esterases:** EstE1 (PDB ID: 2C7B), AFEST (*Archaeoglobus fulgidus*, PDB ID: 1JJI), Sto-EST (*Sulfolobus tokodaii*, PDB ID: 3AIK). **Mesophilic esterases:** EcEst (*Escherichia coli*, NCBI ID: EID7305910.1), rPPE (*Pseudomonas putida*, PDB ID: 4OB8), Est8 (*Parvibaculum* sp., PDB ID: 4YPV). **Psychrotrophic esterases:** EstK (*Pseudomonas mandelii*, UniProt ID: H6VXL7). **Psychrophilic esterases:** EstSP1 (*Sphingomonas glacialis*, NCBI ID: WP_010186968.1), ThaEst2349 (*Thalassospira* sp., PDB ID: 4V2I).

To address this, we investigated how EstE1 achieves its remarkably high catalytic rate, focusing on the potential role of the catalytic His loop in modulating enzyme activity. Structural comparisons revealed that EstE1 contains Gly282 adjacent to catalytic His281, whereas rPPE—sharing 40% sequence identity—has Asp287 at the equivalent position next to His286 (Fig. 1A). In rPPE, Asp287 forms hydrogen bonds with Asn158 (located in the catalytic Ser loop) and Trp187 (in the active-site wall), likely stabilizing the His loop. These stabilizing interactions are absent in EstE1, where instead a contact occurs between Tyr182 (corresponding to Trp187 in rPPE) and the backbone of Val284 within the His loop. Gly at this position is conserved in other hyperthermophilic esterases such as AFEST and Sto-EST (*Sulfolobus tokodaii*) (12, 23), whereas Asp is typically found at the equivalent position in orthologs from lower-temperature environments (Fig. 1B).

Based on these structural observations, we hypothesized that Gly282 contributes to His-loop flexibility in EstE1 and facilitates its high catalytic rate. To test this, we introduced G282N and G282Q mutations in EstE1 to promote hydrogen bond formation with Asp153, mimicking the Asn158-Asp287 interaction in rPPE but in reverse orientation. These mutations were expected to affect activity either by forming new hydrogen bonds or, if unsuccessful, by introducing steric hindrance that could restrict loop flexibility due to the enzyme’s rigid scaffold. In parallel, we generated rPPE D287G to disrupt the native hydrogen bond network, creating an EstE1-like configuration and D287E to potentially strengthen it. These comparative mutations allowed us to investigate how catalytic His-loop flexibility modulates the balance between activity and stability across different thermal environments.

## RESULTS

### Protein expression and purification

Recombinant EstE1 and rPPE WT and mutant proteins were expressed as soluble proteins in *Escherichia coli* BL21(DE3). All proteins were purified to apparent homogeneity using HisTrap nickel-affinity, Capto Q anion-exchange, and Superdex 200 size-exclusion chromatography. SDS-PAGE analysis showed single bands of approximately 35 kDa for EstE1 and 34 kDa for rPPE (Fig. S1). Size-exclusion chromatography confirmed that EstE1 exists as a dimer (93.2 kDa), while rPPE exists as a monomer (46.8 kDa) (Fig. S2), consistent with previous reports (8, 19).

### Enzyme activity

**Fig 2:**
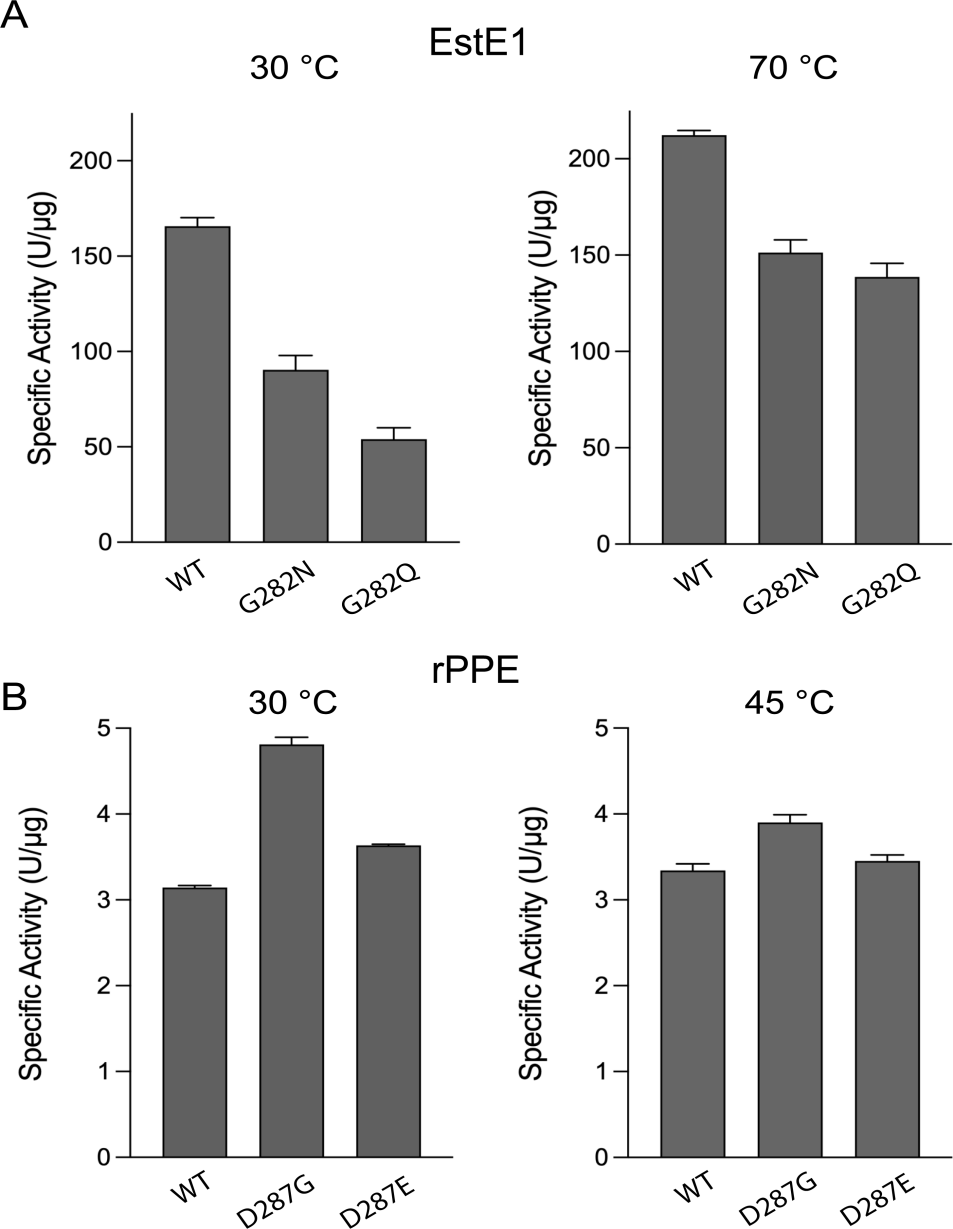
Specific activities of EstE1 and rPPE WT and mutants. (**A**) Specific activities of EstE1 WT and mutants at 30°C and 70°C. (**B**) Specific activities of rPPE WT and mutants at 30°C and 45°C. Enzyme activity was measured for 3 min at the indicated temperatures. Experiments were performed in triplicate; data represent the mean ± SD of three independent experiments.

Enzyme activities were measured at their respective optimal temperatures: 70°C for EstE1 (WT and mutants), 45°C–50°C for rPPE WT, and 30°C for rPPE mutants (Fig. 2). EstE1 activity was also assessed at 30°C to allow direct comparison under identical conditions. At 70°C, EstE1 G282N and G282Q retained 71% and 65% of EstE1 WT activity (215 U/µg), respectively. For rPPE, at 45°C, D287G and D287E exhibited 118% and 109% of rPPE WT activity (3.3 U/µg), respectively. When measured at 30°C, these trends became more pronounced: EstE1 G282N and G282Q activities decreased further to 54% and 32% of WT activity, whereas rPPE D287G and D287E activities increased to 153% and 116% of WT activity, respectively. The reduced activities of EstE1 G282N and G282Q likely reflect restricted His-loop flexibility caused by hydrogen bond formation or steric hindrance introduced by the larger side chains. In contrast, disruption of the stabilizing hydrogen bonds in rPPE D287G enhanced activity, while D287E showed a moderate increase, likely due to hydrophobic exposure despite the formation of a stronger hydrogen bond via Glu287.

### Thermal stability by intrinsic fluorescence

**Fig 3:**
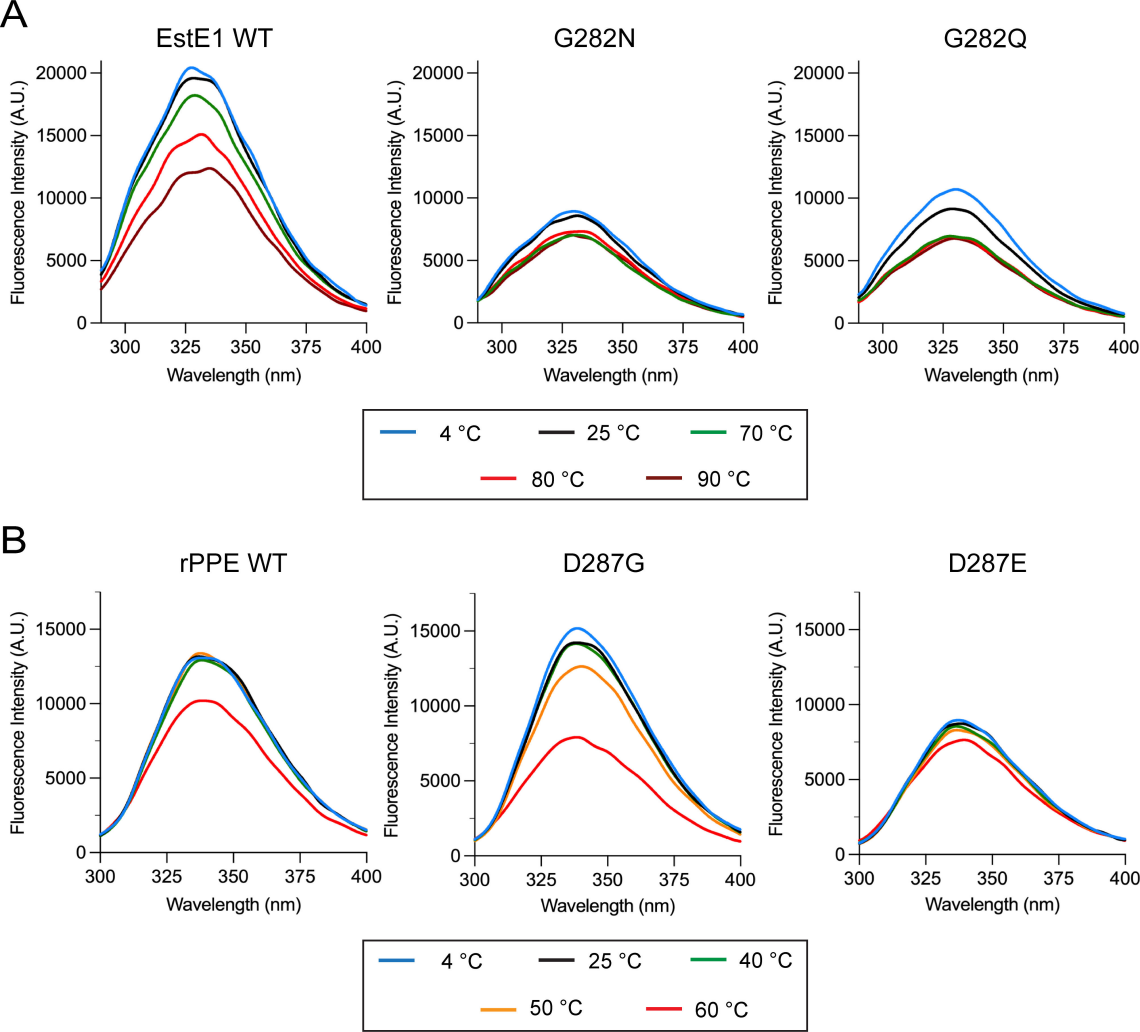
Tertiary structure stability and flexibility of EstE1 and rPPE WT and mutants. Temperature-induced unfolding was analyzed by measuring intrinsic fluorescence after 1 h incubation at various temperatures (4°C–90°C for EstE1 (**A**); 4°C–60°C for rPPE (**B**)) with excitation at 280 nm. Data represent the mean of three independent experiments.

Intrinsic fluorescence was measured after 1 h incubation across temperature ranges (4°C–90°C for EstE1; 4°C–60°C for rPPE). EstE1 WT exhibited a gradual, temperature-dependent fluorescent decrease (Fig. 3A), with Tyr182 and other Tyr residues (Tyr78, Tyr79, Tyr218, and Tyr260) in the hydrophobic regions of the protein contributing to the overall signal. The Y182F mutant showed reduced fluorescence (56% of WT) and a distinct pattern, with similar fluorescence at 4°C–25°C and 70°C–90°C (Fig. S4), indicating that Tyr182 plays a role in catalytic His-loop flexibility in EstE1 WT. G282N showed similar fluorescence across 4°C–25°C and 70°C–90°C but at lower intensity, while G282Q displayed a more pronounced decrease between 4°C and 25°C, maintaining its levels at 70°C–90°C. These results suggest that G282N stabilizes the His loop, whereas G282Q exhibits flexibility similar to WT despite its substitution.

In rPPE, WT fluorescence remained stable up to 50°C but decreased at 60°C (Fig. 3B). D287G remained stable up to 40°C, then declined, retaining ~50% intensity at 60°C. D287E remained relatively stable across 4°C–60°C but exhibited overall lower fluorescence intensity than WT and D287G. The fluorescence decline observed for D287G suggests increased flexibility in the active-site region, while D287E appears more rigid. Thus, EstE1 maintains local active-site flexibility despite its global rigidity, whereas rPPE exhibits local rigidity in the active site despite having a more globally flexible scaffold.

### Active-site stability

**Fig 4:**
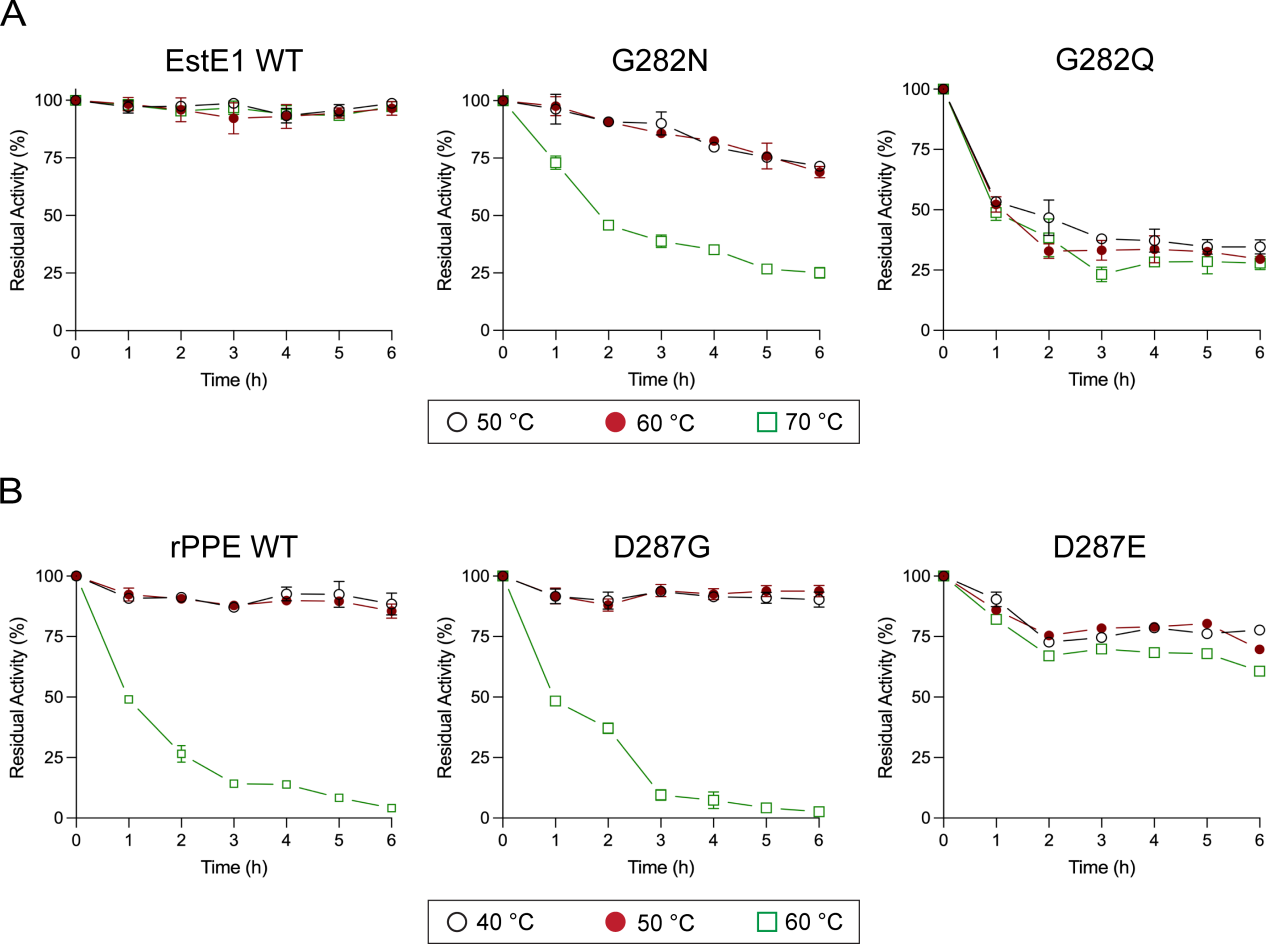
Thermal stability of EstE1 and rPPE WT and mutants. (A) EstE1 WT and mutants were incubated at 50°C, 60°C, and 70°C for up to 6h. (B) rPPE WT and mutants at 40°C, 50°C, and 60°C for up to 6 h. Residual activity was measured at the optimum temperature. Activity before incubation was set to 100%. Experiments were performed in triplicate; data represent mean ± SD of three independent experiments.

Residual activities were measured at each enzyme’s optimal temperature after pre-incubation at elevated temperatures (50°C–70°C for EstE1; 40°C–60°C for rPPE) (Fig. S3). EstE1 WT remained stable across the entire temperature range, while G282N gradually lost activity at 50°C–60°C, retaining only 25% activity at 70°C. G282Q was more heat-sensitive, maintaining ~30% activity across all temperatures tested (Fig. 4A). In rPPE, WT, D287G, and D287E were stable at 40°C–50°C (Fig. 4B). However, at 60°C, WT and D287G showed substantial activity loss, while D287E retained higher residual activity. These findings indicate that Gly282 in EstE1 helps maintain active-site integrity by minimizing steric interference within the His loop, while Glu287 in rPPE enhances stability by forming a stronger hydrogen bond.

### Conformational flexibility

**Fig 5:**
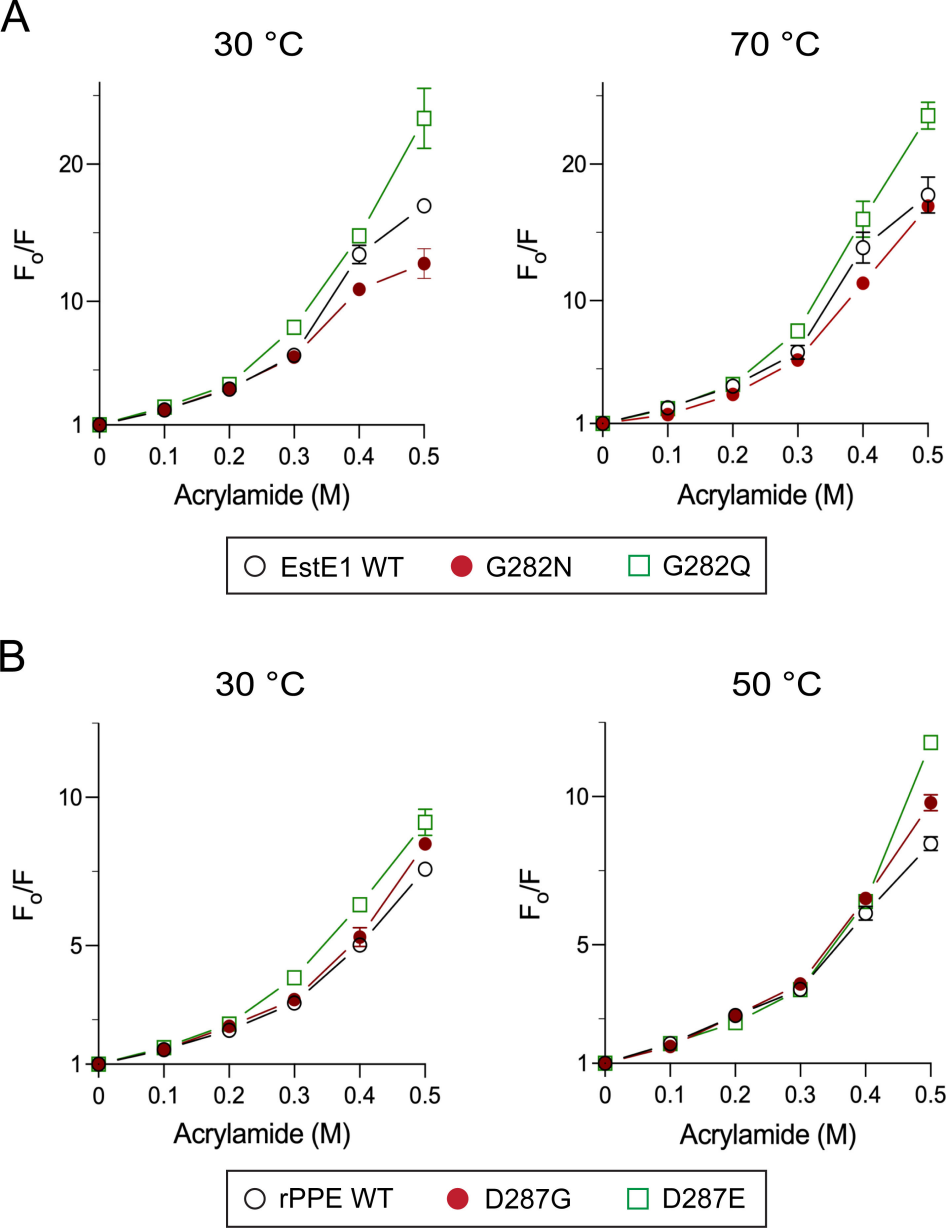
Structural flexibility of EstE1 and rPPE by fluorescence quenching. (**A**) Structural flexibility of EstE1 at 30°C and 70°C. (**B**) Structural flexibility of rPPE at 30°C and 50°C. Stern–Volmer plots were generated by recording maximum fluorescence intensities (*F*_0_*/F*) at increasing acrylamide concentrations (0–0.5 M) upon excitation at 280 nm. *F*_0_ and *F* represent fluorescence intensities in the absence and presence of acrylamide, respectively. Data represent the mean ± SD of three independent experiments.

Acrylamide-induced quenching was used to assess conformational flexibility at 30°C and each enzyme’s optimal temperature (70°C for EstE1; 50°C for rPPE). In EstE1, fluorescence intensity and quenching profiles followed the trend G282N < WT < G282Q at both temperatures (Table S2; Fig. 5A). In rPPE, flexibility increased at 50°C compared to at 30°C, following WT < D287G < D287E (Table S2; Fig. 5B), with the largest differences observed at 0.5 M acrylamide. The similar fluorescence levels of EstE1 WT at 30°C and 70°C likely reflect contributions from Tyr182 (Fig. 3A). G282N increases active-site rigidity, while G282Q fails to form a stabilizing interaction. In rPPE, removal of the Asp287-mediated hydrogen bond in D287G increases flexibility, while D287E shows unexpectedly higher flexibility despite the formation of a stronger hydrogen bond, likely due to exposure of hydrophobic regions, as suggested by its lower intrinsic fluorescence (Fig. 3B).

### Secondary structure analysis

**Fig 6:**
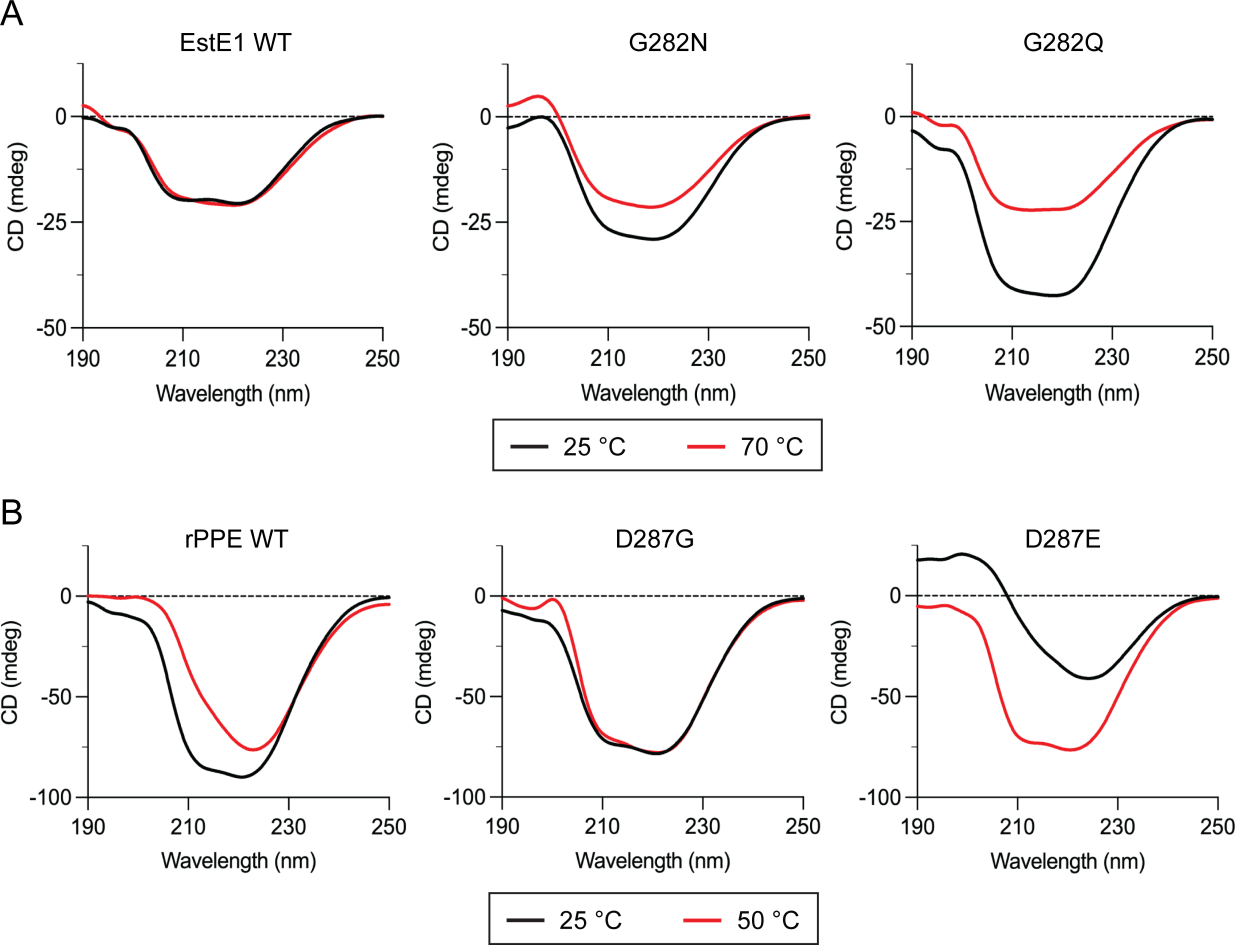
Far-UV CD spectra of EstE1 and rPPE WT and mutants. (**A**) CD spectra of EstE1 at 25°C and 70°C. (**B**) CD spectra of rPPE at 25°C and 50°C. Enzyme samples (0.36 mg/mL) were incubated at the indicated temperatures for 1 h and measured at 25°C.

Far-UV circular dichroism (CD) spectra were recorded after 1 h incubation at 25°C and elevated temperatures (70°C for EstE1; 50°C for rPPE) (Fig. 6). EstE1 WT retained its secondary structure, with α-helix content decreasing from 30% at 25°C to 25% at 70°C (Table S3). G282N and G282Q showed higher α-helix content at 25°C (37% and 49%, respectively) but similar levels to WT at 70°C. These results suggest that the intended hydrogen bonds with Asp153 may not have formed as designed and that alternative structural effects such as compensatory interaction or steric hindrance may have contributed to the altered flexibility observed. In rPPE, WT α-helix content decreased from 25% at 25°C to 6% at 50°C. D287G maintained ~28% α-helix at both temperatures, while D287E had lower content at 25°C but similar levels to WT at 50°C. Notably, EstE1 WT and rPPE D287G—both containing Gly in the catalytic His loop—exhibited minimal temperature-dependent structural changes. These results suggest that the mutations primarily affect secondary structure at 25°C, when proteins are more rigid, while elevated temperatures promote conformational adjustments that maintain overall structure.

### Enzyme kinetics

Steady-state kinetic parameters were determined at optimal temperatures (Table 1). EstE1 WT showed a Michaelis constant ($K_{m}$) of 1,520 µM and a catalytic rate (_^$k_{cat}$^_) of 2,900 s⁻¹. The G282N mutant exhibited slightly improved substrate affinity ($K_{m}$ 1,330 µM) but reduced $k_{cat}$ (1,980 s⁻¹), whereas G282Q displayed the highest affinity ($K_{m}$ 745 µM) and a markedly reduced $k_{cat}$ (950 s⁻¹), resulting in the lowest catalytic performance. In rPPE, WT showed a $K_{m}$ of 33 µM and $k_{cat}$ of 2.0 s⁻¹. D287G increased $K_{m}$ (3.5-fold) and $k_{cat}$ (6.5-fold), yielding a 1.8-fold increase in specificity constant $\left(k_{cat}/K_{m}\right)$. D287E significantly reduced $K_{m}$, while slightly increasing $k_{cat}$, resulting in a 5.5-fold increase in specificity constant. These data suggest that removal of the hydrogen bonds increases flexibility and turnover but weakens substrate binding, whereas formation of a stronger hydrogen bond improves binding affinity but slightly reduces turnover.

**TABLE 1:** Kinetic parameters of EstE1 and rPPE at their optimal temperatures^*a*^

		*K*_m_ (μM)	*k*_cat_ (s^−1^)	*k*_cat_/*K*_m_ (s^−1^μM^−1^)
EstE1	WT	1520 ± 17	2900 ± 1	1.91 ± 0.02
	G282N	1330 ± 79	1980 ± 84	1.49 ± 0.03
	G282Q	745 ± 40	950 ± 27	1.28 ± 0.03
rPPE	WT	33 ± 1	2.0 ± 0.1	0.06 ± 0.01
	D287G	114 ± 2	13 ± 1	0.11 ± 0.01
	D287E	6 ± 1	2.1 ± 0.1	0.33 ± 0.02

^*a*^ Data are presented as mean ± SD from three independent experiments.

### MD simulations

**Fig 7:**
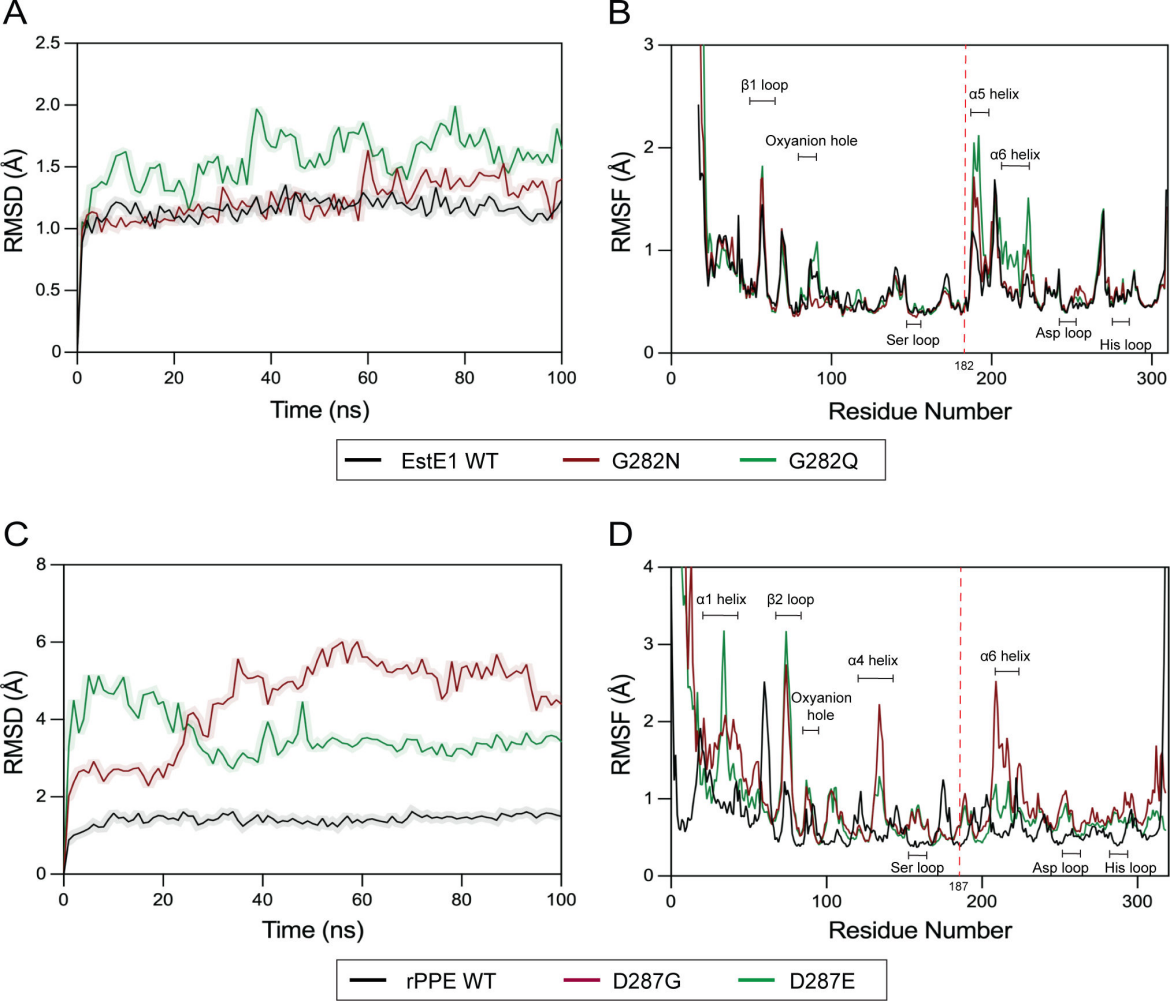
MD simulations of EstE1 and rPPE WT and mutants. (**A and B**) EstE1: (**A**) RMSD plots and (**B**) RMSF plots of C_α_ atoms. (**C and D**) rPPE: (**C**) RMSD plots and (**D**) RMSF plots of C_α_ atoms.

Molecular dynamics (MD) simulations were performed to assess structural impacts of the mutations. EstE1 WT and G282N reached steady-state within 60 ns, maintaining C_α_-root-mean-square deviation (RMSD) values around 1.2 Å (Fig. 7A). G282N stabilized approximately 0.3 Å higher than WT, suggesting a subtle conformational shift. G282Q showed the highest RMSD (1.6 Å), indicating greater fluctuation. Both EstE1 mutants exhibited increased flexibility in the β1 loop (residues 56–57), α5 helix (192–201), and α6 helix (207–219) (Fig. 7B). In the oxyanion hole, G282N slightly reduced fluctuation, while G282Q increased it. Tyr182 remained stable in both mutants.

In rPPE, WT stabilized rapidly with an RMSD of 1.5 Å. D287G and D287E reached equilibrium after 30 ns, with RMSD values ranked WT (1.5 Å) <D287E (3.4 Å) <D287G (5.6 Å) (Fig. 7C). Both mutants exhibited increased flexibility, particularly in the α1 helix, β2 loop, and oxyanion hole. D287G showed pronounced fluctuations in the α4 and α6 helices, with elevated C_α_-root-mean-square fluctuation (RMSF) values at Trp187 in both mutants (Fig. 7D). D287G exhibited the greatest flexibility, consistent with loss of the stabilizing hydrogen bonds. D287E had slightly reduced fluctuations but remained more flexible than WT. These results indicate that His-loop mutations affect global structural dynamics, with altered flexibility observed in the oxyanion hole and α6 helix in both enzymes, and additional shifts in the β1 loop and α5 helix (EstE1) and α1 helix, β2 loop, and α4 helix (rPPE).

## DISCUSSION

Enzyme thermal stability is often associated with reduced flexibility, which can lower catalytic turnover (15, 24–26). Excessive rigidity limits essential conformational changes required for catalysis, while excessive flexibility compromises structural integrity at high temperatures (22, 27–29). This balance between stability and activity, commonly referred to as the activity-stability trade-off, has been challenged by studies of thermostable esterases, particularly those developed through directed evolution (30). Subsequent investigations have demonstrated that this trade-off does not universally apply, even for hyperthermophilic enzymes (31). Our findings suggest that EstE1 achieves high catalytic turnover by incorporating local flexibility within the catalytic His loop while maintaining an otherwise globally rigid structure. This strategy enables both high activity and exceptional thermal stability. The altered fluorescence profile of the Y182F mutant underscores Tyr182 as a key contributor to this flexibility. This observation is consistent with the psychrophilic esterase EstK, where Trp208 at the equivalent position dominates fluorescence among multiple Trp residues (30). The gradual, temperature-dependent fluorescence decline observed in EstE1 WT likely reflects localized conformational changes near the catalytic site, particularly involving Gly282-dependent His-loop flexibility, rather than global unfolding. Substituting Tyr182 with Ala reduced activity but had little effect on thermal stability (21), which is consistent with the rigid scaffold characteristic of hyperthermophilic enzymes (32, 33). In contrast, in rPPE, the Trp187-Asp287-Asn158 hydrogen bond network stabilizes the catalytic His loop at lower temperatures, as evidenced by stable fluorescence at 4°C and 25°C.

The preference for aromatic residues at this active-site position appears to correlate with habitat temperature (30, 34–36). Hyperthermophilic EstE1 favors Tyr182, mesophilic rPPE favors Trp187, psychrotrophic EstK favors Trp208, and psychrophilic EstSP1 favors Tyr191, all occupying equivalent positions within the active-site wall. Hyperthermophilic and psychrophilic esterases tend to favor Tyr, whose hydroxyl group forms stabilizing hydrogen bonds (34, 35), compensating for reduced hydrophobicity of aromatic residues at extreme temperatures (37). Substituting Tyr182 with Trp in EstE1 caused structural instability due to steric hindrance from the bulky Trp side chain (21). Conversely, mesophilic and psychrotrophic esterases, which experience larger temperature fluctuations, tend to favor Trp because of its ability to provide both hydrophobic stabilization and hydrogen bonding. These observations suggest that Tyr182 supports active-site flexibility in EstE1, while Trp187 contributes to greater rigidity in rPPE’s active site.

**Fig 8:**
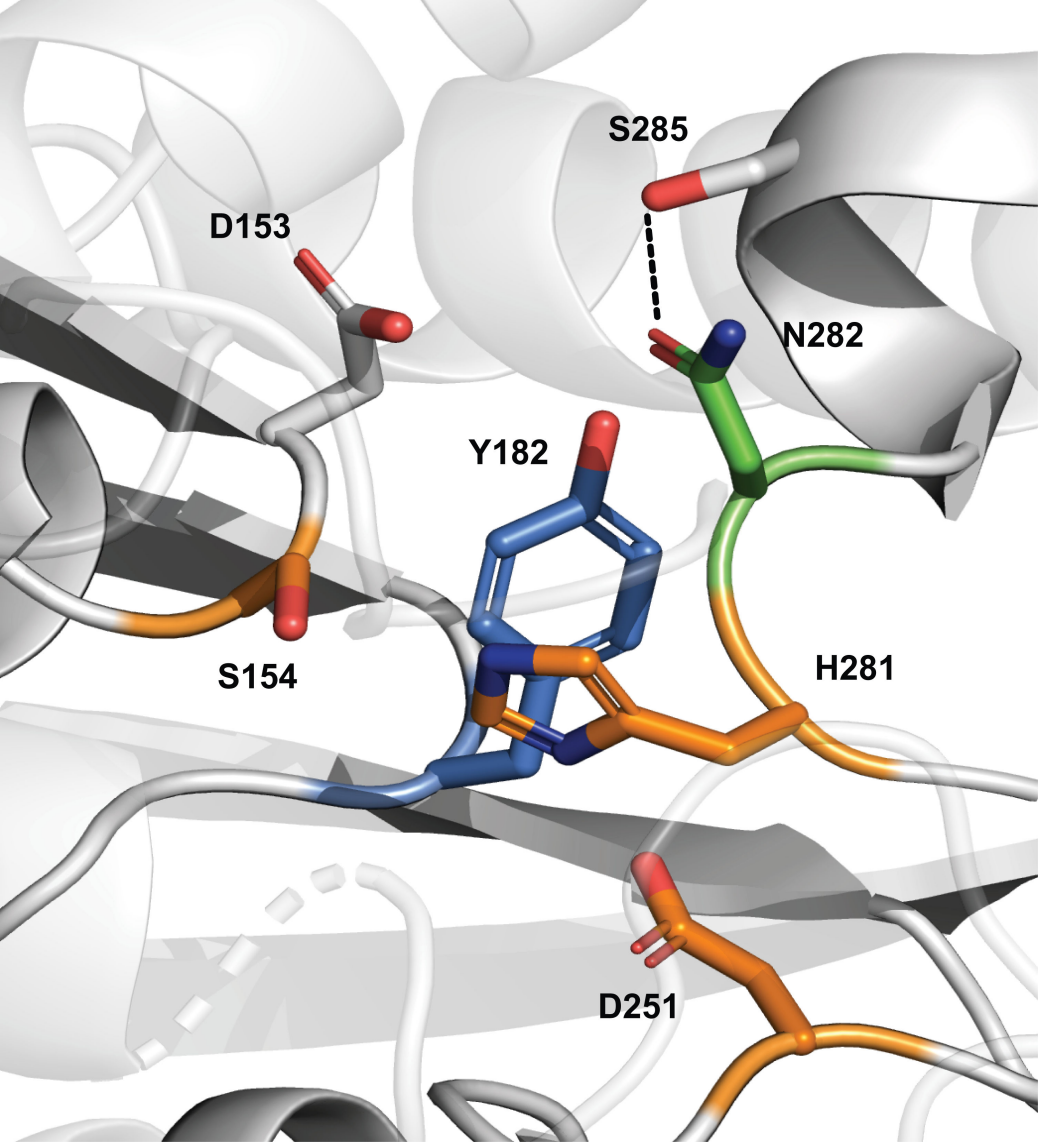
Structural model of EstE1 G282N generated by AlphaFold2 and visualized with PyMOL.

Certain active-site sequence motifs, such as Y–X–Gly or Gly–X–Y, where X and Y are small, low-polarity residues, minimize steric interference while maintaining stability (38). In EstE1, the Val-His-Gly282-Phe-Val sequence includes Gly, which lacks a side chain, and small Val residues that support loop flexibility. Gly282 thus plays a crucial role in preserving His-loop flexibility. Structural modeling revealed that in G282N, Asn282 failed to form the intended hydrogen bond with Asp153 but instead formed an unintended hydrogen bond with Ser285 (Fig. 8), partially restricting loop mobility. In G282Q, the longer Gln side chain not only failed to form the intended bond but also introduced steric hindrance, disrupting the active-site environment and further reducing activity and stability.

In rPPE, removal of the Asp287 hydrogen bonds (D287G) expanded the substrate-binding pocket, enhancing activity but reducing stability. MD simulations confirmed greater flexibility in D287G than in WT and D287E (Fig. 7), consistent with its decreased stability. The D287E mutant, despite forming a stronger hydrogen bond, showed unexpectedly increased flexibility, likely due to hydrophobic exposure. Although CD indicated substantial secondary structure shifts, these aligned with structural fluctuations observed in MD simulations and fluorescence analyzes, indicating broader changes in D287E. These changes also improved substrate affinity and specificity constant. While direct structural confirmation of loop hydrogen bonding is unavailable, biophysical and kinetic data support altered local interactions in both EstE1 and rPPE mutants.

A similar mechanism is observed in EstK, where substituting Asp308 with Ala enlarged the substrate-binding pocket, increasing activity but reducing stability—reflecting a preference for stability over activity in cold-adapted, inherently flexible enzymes. Additionally, both EstE1 and rPPE possess a hydrogen bond network (Tyr78-His92-Asp153 in EstE1; His97-Asn158 in rPPE) that stabilizes the catalytic Ser loop and oxyanion hole (10). Together, these findings suggest that EstE1 and its lower-temperature orthologs use both shared and distinct strategies to stabilize their active sites while balancing catalytic activity and structural stability.

Additional structural features also contribute to overall enzyme integrity. A mid-region salt bridge between α8 and β8 (Glu258–Arg275 in EstE1; Glu263–Arg280 in rPPE) helps secure the catalytic Asp (β7 loop) and His (β8 loop) (30, 34, 36). The C-terminal β8–α9 region plays a similarly important role: in EstE1, hydrophobic interactions between Phe276 and Leu299 reinforce dimer stability, while in monomeric rPPE, a hydrogen bond between Tyr281 and Gln306 stabilizes this region (19). Disrupting either interaction increased local flexibility in EstE1 without substantially compromising thermal stability, likely due to its robust scaffold. In contrast, similar disruptions in rPPE caused marked destabilization. Beyond His-loop composition, differences in oligomeric state and folding architecture between dimeric EstE1 and monomeric rPPE likely contribute to catalytic behavior independently of His-loop mutations.

In addition to their effects on turnover, localized flexibility facilitates substrate binding and transition-state stabilization, influencing substrate preferences across thermal environments (19, 21, 30, 34, 39). Hyperthermophilic esterases, with flexible active sites embedded in rigid scaffolds, preferentially hydrolyze pNP esters with C4 and C6 chains (19, 22, 34, 40). In contrast, mesophilic rPPE and psychrotrophic EstK, which stabilize active sites through His-loop interactions within more flexible overall structures, favor pNP esters with shorter C2 chains that require lower activation energy for hydrolysis (19, 30).

In summary, these findings provide mechanistic insights into how hyperthermophilic esterases achieve high catalytic activity while maintaining structural rigidity. EstE1 incorporates local active-site flexibility through its catalytic His loop, while its lower-temperature orthologs rely on hydrogen bonding to stabilize the His loop within a more globally flexible scaffold. These insights offer a framework for engineering esterases with enhanced catalytic performance and stability.

## MATERIALS AND METHODS

### Materials

The pET28 expression vector was obtained from Novagen (Madison, WI, USA). The *este1* plasmid was provided by Dr. Jong-Won Oh (Yonsei University, Seoul, South Korea), while the *rppe* gene was synthesized by GenScript (Piscataway, NJ, USA). The EZchange site-directed mutagenesis kit and nPfu polymerase were purchased from Enzynomics (Daejeon, South Korea). Protein purification columns, including HisTrap, HiTrap desalting, Capto Q, and Superdex 200 Prep Grade XK16, were obtained from Cytiva (Marlborough, MA, USA). pNP acetate and pNP hexanoate were purchased from Sigma-Aldrich (St. Louis, MO, USA). All other chemicals were obtained from Sigma-Aldrich or Tokyo Chemical Industry (Tokyo, Japan).

### Sequence and structure analyses

The crystal structures of EstE1 (PDB ID: 2C7B) and rPPE (PDB ID: 4OB8) were visualized using PyMOL (The PyMOL Molecular Graphics System, version 2.0 Schrödinger, LLC, New York, NY, USA). Structural models were generated using AlphaFold2, incorporating data from the AlphaFold Protein Structural Database (41, 42). Model confidence was assessed using the predicted local distance difference test (pLDDT) score, where pLDDT >95 indicates high structural reliability (41, 42). Multiple sequence alignments of esterases adapted to different thermal environments were performed using Clustal Omega (43).

### Site-directed mutagenesis

Site-directed mutagenesis was performed to introduce mutations in EstE1 (G282N and G282Q) and rPPE (D287G and D287E) using the EZchange site-directed mutagenesis kit, with primers listed in Table S1. PCR amplification was performed with nPfu polymerase, followed by DpnI digestion to eliminate parental plasmids. Mutant constructs were confirmed by Sanger sequencing.

### Expression and purification of EstE1 and rPPE

Recombinant WT and mutant EstE1 and rPPE proteins were expressed in *E. coli* BL21(DE3) grown in LB medium. Protein expression was induced with 1 mM isopropyl β-D-1-thiogalactopyranoside when cultures reached an OD_600_ of 0.6–0.8, followed by incubation at 25°C for 18 h. Cells were harvested by centrifugation (6,000 × *g*, 15 min), resuspended in buffer A (50 mM Tris·HCl, 50 mM NaCl, 5 mM imidazole, pH 8.0), and lysed by sonication. Lysates were clarified by centrifugation (10,000 × *g*, 20 min, 4°C), and the supernatant was applied to a 1 mL HisTrap nickel-affinity column. Proteins were purified with a step gradient of 50 mM and 300 mM imidazole in buffer A. Pooled fractions were further purified by Capto Q anion-exchange chromatography on an AKTA go system (Cytiva), equilibrated with buffer C (50 mM Tris·HCl, pH 7.5), and eluted with a linear gradient of 50–1,000 mM KCl in buffer D (50 mM Tris·HCl, 1,000 mM KCl, pH 7.5). Final purification and molecular weight determination were performed by size-exclusion chromatography using a Superdex 200 column equilibrated with buffer E (50 mM sodium phosphate, 150 mM NaCl, pH 7.0). Purified proteins were desalted using a HiTrap desalting column, pooled in reaction buffer (100 mM Tris·HCl, 100 mM NaCl, pH 7.5), containing 5% glycerol, and stored at −80°C. Protein concentrations were determined by the Bradford assay with bovine serum albumin as the standard.

### Enzyme activity and kinetic analysis

Enzymatic activity was measured using pNP hexanoate (2.5 pmol) for EstE1 and pNP acetate (100 pmol) for rPPE in reaction buffer. Absorbance changes at 405 nm were recorded for 3 min using a Shimadzu UV-1800 spectrophotometer (Kyoto, Japan), with background hydrolysis subtracted. One unit of enzyme activity was defined as the amount required to hydrolyze 1 pmol of pNP substrate per min at the respective optimal temperatures. Optimal temperatures were determined by measuring activity across a range of 30°C–90°C for EstE1 and 20°C–60°C for rPPE. Thermal stability was assessed by incubating enzymes at different temperatures for up to 6 h, with activity measured at 1 h intervals under optimal conditions. The activity before incubation was set to 100% as a reference. Steady-state kinetic parameters (*K*_m_, *V*_max_, and *k*_cat_) were determined from Lineweaver-Burk plots.

### Fluorescence spectroscopy analyses

Conformational flexibility was assessed by acrylamide-induced fluorescence quenching using a Scinco FS-2 fluorescence spectrometer (Seoul, South Korea) at room temperature. Protein samples (10  µM) in reaction buffer were incubated with acrylamide (0–0.5 M) at 30°C for 3 min prior to measurement. Fluorescence was recorded with excitation at 280 nm and emission spectra collected between 290 and 400 nm. Quenching data were analyzed using GraphPad Prism and expressed as *F*_0_/*F* ratios, where *F*_0_ is the fluorescence without acrylamide and *F* is the fluorescence at increasing acrylamide concentrations (0.1–0.5 M) (44). The Stern–Volmer equation was used:


$$F_{0}/F\;=1+\;K_{SV}\left[Q\right]$$

where *K*_sv_ is the Stern-Volmer constant and [*Q*] is the acrylamide concentration. The slope of the quenching curve was used to calculate *K*_sv_ (45).

### Far-UV CD spectroscopy

Secondary structure changes were analyzed by far-UV CD spectroscopy (190–250 nm) using a JASCO J-1500 spectropolarimeter (Kyoto, Japan) at the Korea Basic Science Institute (Ochang, South Korea). Protein samples (0.36 mg/mL) were incubated at 25°C and their respective optimal temperatures (70°C for EstE1 and 50°C for rPPE) for 1 h prior to measurement. The α-helix content was estimated using the BeStSel server (46) for 1 h prior to measurement. The α-helix content was estimated using the BeStSel server.

### MD simulation

MD simulations were performed using Maestro 12.0 (Schrödinger, LLC, New York, NY, USA) with the SPC solvent model and OPLS4 force field. Protein structures were prepared using the Protein Preparation Wizard by removing water molecules and optimizing the geometry. Water molecules were replaced with ions as necessary to maintain system neutrality. Energy minimization was carried out for 1,000 steps to resolve steric clashes. Simulations were performed at BioCode Ltd. (Liverpool, Merseyside, UK) and run for 100 ns, iteratively calculating atomic forces, positions, and velocities to model molecular motion over time (47, 48). Proteins were equilibrated in an NTP ensemble at 300 K, and the resulting trajectory provided atomic-level configurations for analysis throughout the simulation.

## Data Availability

The data that support the findings of this study are available from the corresponding author upon reasonable request.

## SUPPLEMENTAL MATERIAL

Table S1 to S3 and Figures S1 to S4 spectrum.01003-25-s0001.pdf List of PCR primers, Inverse Stern-Volmer quenching constant, Protein secondary structure analysis, SDS-PAGE analysis, Size-exclusion chromatography analysis, Optimal temperatures, and Tertiary structure stability of EstE1 Y182F.

ASM does not own the copyrights to Supplemental Material that may be linked to, or accessed through, an article. The authors have granted ASM a non-exclusive, world-wide license to publish the Supplemental Material files. Please contact the corresponding author directly for reuse.
