# Supplementary material for: Catalytic His-loop flexibility drives high activity in hyperthermophilic esterase EstE1 while preserving structural stability
Source: Microbiol Spectr. 2025 Aug 14;13(10):e01003-25. doi: 10.1128/spectrum.01003-25 (PMC12502808; doi:10.1128/spectrum.01003-25)
Supplement: Table S1 to S3 and Figures S1 to S4 — List of PCR primers, Inverse Stern-Volmer quenching constant, Protein secondary structure analysis, SDS-PAGE analysis, Size-exclusion chromatography analysis, Optimal temperatures, and Tertiary structure stability of EstE1 Y182F. [file spectrum.01003-25-s0001.pdf]

Supporting Information for:

Catalytic His-loop flexibility drives high activity in hyperthermophilic esterase EstE1 while preserving structural stability

Khang Nguyen,[a] ChangWoo Lee [a, *]

[a]Department of Biomedical Science and Center for Bio-Nanomaterials, Daegu University, Gyeongsan 38453, South Korea

*Corresponding Author: Department of Biomedical Science, Daegu University, Gyeongsan 38453, South Korea, E-mail: leec@daegu.ac.kr, Tel.: +82-53-850-6464

Supporting Table S1-S3

Supporting Figure S1-S4

Table S1. List of PCR primers for site-directed mutagenesis.

	Primer sequence
EstE1^{G282N}	5′-GGGATGGTCCACAACTTTGT-3′ and
	5′-GAAGCTGACAAAGTTGTGGAC-3′
EstE1^{G282Q}	5′-GGGATGGTCCACCAGTTTGT-3′ and
	5′-GAAGCTGACAAACTGGTGGAC-3′
EstE1^{Y182F}	5′-GTTGATCTTCCCCGTCGTCAATATGAC-3′ and
	5′-CGACGGGGAAGATCAACACTTGTTTC -3′
rPPE^{D287G}	5′-CGGGATGATCCATGGATATG-3′ and
	5′-GAGCAGCCCATATCCATG-3′
rPPE^{D287E}	5′-CGGGATGATCCATGAGTATG-3′ and
	5′-GAGCAGCCCATACTCATG-3′

Underlined capital letters indicate the codon for the substituted amino acid.

Table S2. Inverse Stern−Volmer quenching constant (K_{SV}^{-1}) of EstE1 and rPPE WT and mutants.

		K_{SV}^{-1} (mM)		
		30 °C	50 °C	70 °C
EstE1	WT	31 ± 1		30 ± 3
	G282N	43 ± 4		31 ± 1
	G282Q	22 ± 2		22 ± 1
rPPE	WT	76 ± 2	68 ± 2	
	D287G	67 ± 1	57 ± 2	
	D287E	62 ± 3	46 ± 1	

Values represent means ± SD from three independent experiments.

Table S3. Protein secondary structure analysis based on far-UV CD spectra.

	Temperature (°C)	Helix (%)	Strand (%)	Turn (%)	Other (%)	Reference
EstE1 WT		36	29	12	23	PDB ID: 2C7B
EstE1 WT	25	30	30	10	30	This study
	70	25	38	9	28	
EstE1 G282N	25	37	54	4	5	
	70	26	31	8	35	
EstE1 G282Q	25	49	51	0	0	
	70	30	35	9	26	
rPPE WT		37	19	12	32	PDB ID: 4OB8
rPPE WT	25	25	61	2	12	This study
	50	6	63	6	25	
rPPE D287G	25	27	50	5	18	
	50	28	51	5	16	
rPPE D287E	25	3	42	12	43	
	50	29	49	5	17	

α-Helix and β-strand content were calculated using the BeStSel server.

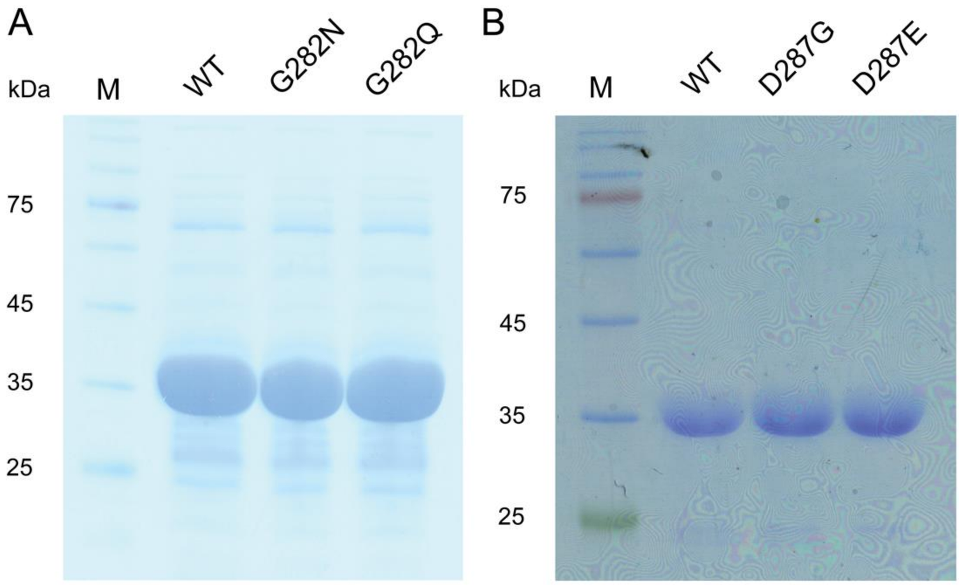

Fig. S1. SDS-PAGE analysis of WT and mutants EstE1 (A) and rPPE (B). M, marker.

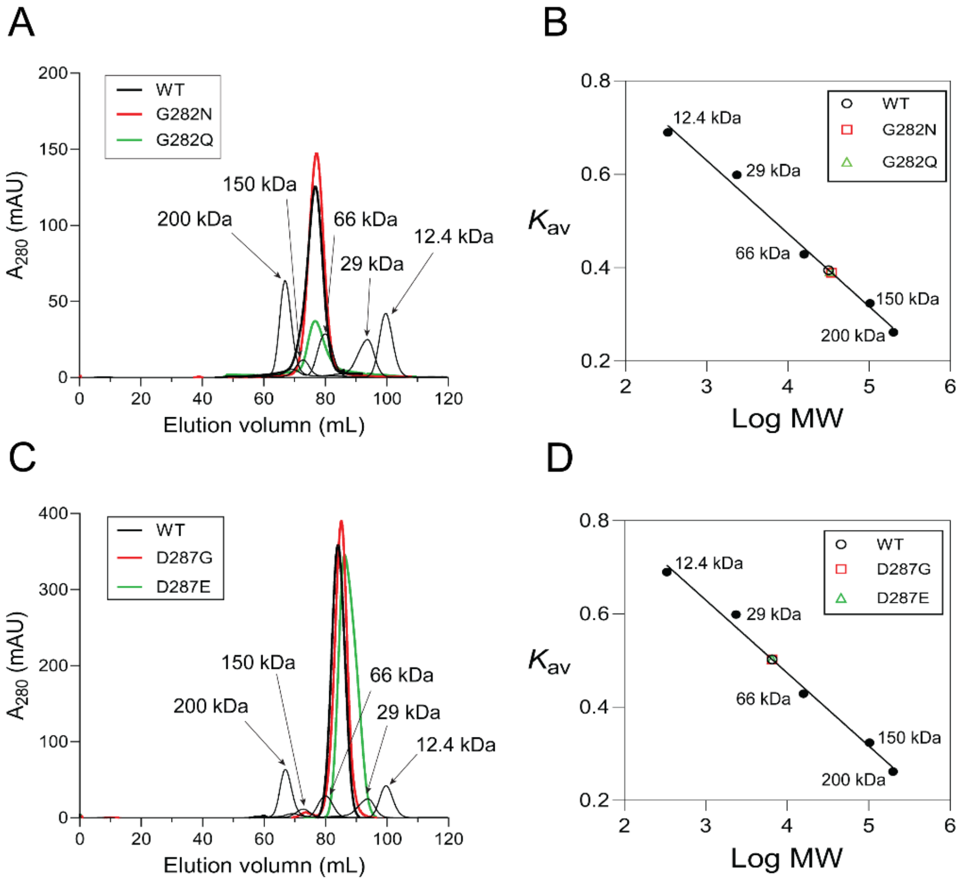

Fig. S2. Size-exclusion chromatography analysis. Protein molecular weights (MWs) were determined using Superdex 200 size-exclusion chromatography in buffer E (50 mM sodium phosphate, 150 mM NaCl, pH 7.0). MWs were analyzed for EstE1 WT and mutants (A, B) and rPPE WT and mutants (C, D). Black lines represent protein MW standards: β-amylase (200 kDa), alcohol dehydrogenase (150 kDa), albumin (66 kDa), carbonic anhydrase (29 kDa), and cytochrome C (12.4 kDa).

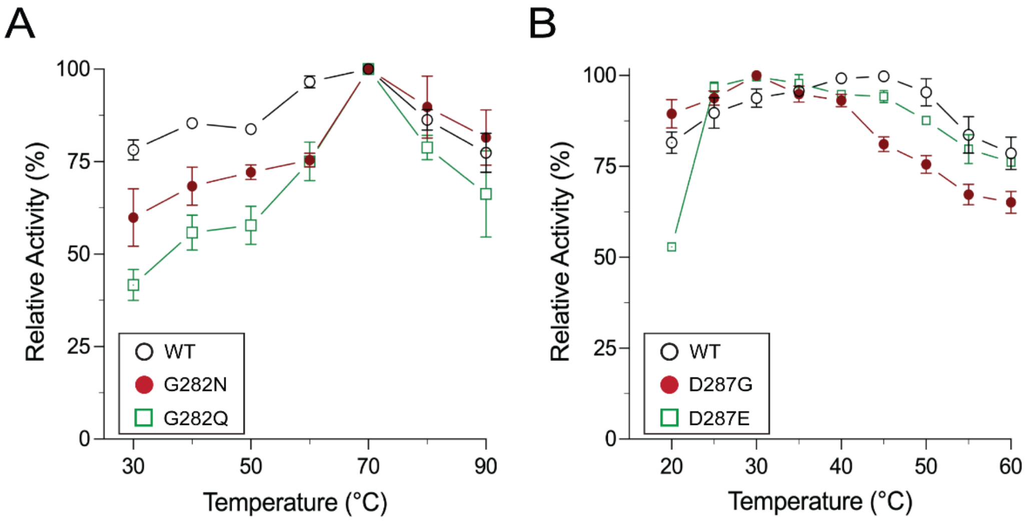

Fig. S3. Optimal temperatures of EstE1 WT and mutants (A) and rPPE WT and mutants (B). Data represent mean ± SD from three independent experiments.

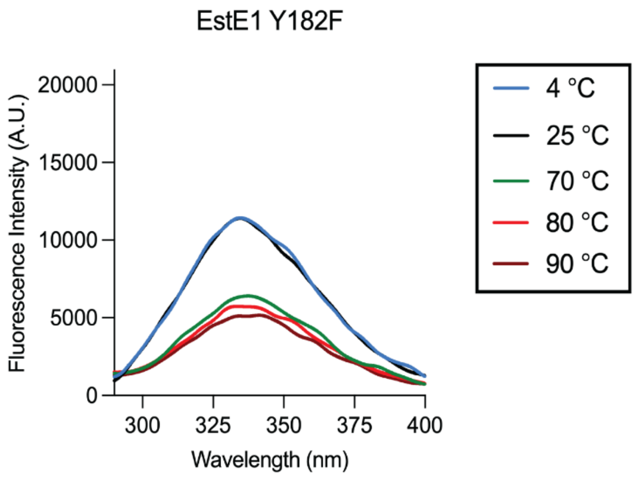

Fig. S4. Tertiary structure stability of EstE1 Y182F. Temperature-induced unfolding was analyzed by measuring intrinsic fluorescence after incubation at various temperatures (4–90 °C) for 1 h (excitation at 280 nm). Data represent the mean of three independent experiments.
